# Hypertensive emergency presenting as blurry vision in a patient with hypertensive chorioretinopathy

**DOI:** 10.1186/s12245-015-0063-6

**Published:** 2015-04-23

**Authors:** Andrew W Stacey, Cemal B Sozener, Cagri G Besirli

**Affiliations:** Department of Ophthalmology and Visual Sciences, University of Michigan, 500 S State St, Ann Arbor, MI 48109 USA; Department of Emergency Medicine, University of Michigan, 500 S State St, Ann Arbor, MI 48109 USA

**Keywords:** Hypertensive emergency, Hypertensive retinopathy, Hypertensive choroidopathy, Fundus photography

## Abstract

A 42-year-old man presented with 3 weeks of blurry vision in the right eye. His exam was significant for decreased vision in the right eye, diffuse retinopathy in both eyes, and serous retinal detachment in the right eye. The patient was found to be hypertensive with blood pressure of 256/160 mmHg. He was diagnosed with hypertensive emergency with end-organ damage due to features of hypertensive chorioretinopathy. He was admitted with abnormal urinalysis, elevated troponin, and abnormal EKG. After appropriate control of his blood pressure, his vision and his labs normalized. Hypertensive emergencies can be manifested first in the eyes. When the choroid is associated, the hypertensive event is often more acute and associated with increased morbidity. It is imperative to obtain a fundus exam in any patient with elevated blood pressure and concomitant vision complaints.

## Background

Systemic hypertension affects 78 million adults in the United States, with only 53% of those carrying this diagnosis achieving appropriate control [1]. Ocular manifestations of systemic hypertension are common and result in vascular compromise at the level of the retina, the choroid, or the optic nerve. The Beaver Dam Eye Study estimated that 10.7% of hypertensive patients over the age of 40 have hypertensive retinopathy and over a 5-year follow-up period, 6% of those who had normal retinal exams developed hypertensive retinopathy [2].

Hypertensive retinopathy is the most common ocular sign of hypertension and is a result of the breakdown of the inner blood-retinal border. It is characterized by retinal arteriolar attenuation, so-called copper wiring of the retinal arterioles caused by sclerosis and hyalinization of the vascular wall, arteriovenous crossing changes (‘nicking’), cotton-wool spots, and in advanced cases, retinal arteriolar exudation leading to a macular star [3]. Hypertensive optic neuropathy is less common and occurs when retinal ischemia causes swelling of the retinal ganglion cells. These cells travel from the inner retina and combine to form the optic nerve. Swelling in the optic nerve leads to optic nerve head elevation and flame-shaped hemorrhages [3].

Hypertensive choroidopathy, seen in combination with retinopathy and also known as hypertensive chorioretinopathy, is caused by fibrinoid necrosis of the choroidal arterioles and is characterized by hypoperfusion of the choriocapillaris (best seen with fluorescein angiography), break down of the retinal pigment epithelial cells causing hypopigmented lesions (Elschnig spots), and subsequent serous retinal detachments [4]. Hypertensive choroidopathy is an uncommon manifestation of hypertension seen in young patients secondary to an acute increase in blood pressure [5]. Its predilection for younger patients is not well understood but thought to be related to elasticity and pliability of younger blood vessels [6]. The choroid is uniquely susceptible to acute rises in blood pressure due to the structure of this vascular network. The vessels of the choriocapillaris branch at right angles, leaving them more susceptible to acute blood pressure changes than the more acute branching vessels of the retina [5,7]. In addition, the choriocapillaris is regulated by the sympathetic nervous system. Acute rises in blood pressure in the choroidal vessels, therefore, will cause counterproductive vasoconstriction and earlier when compared to vessels in the retina [5,7]. The dramatic findings of hypertensive chorioretinopathy are associated with poor visual and systemic prognosis due to their association with systemic illnesses seen in young patients with acute rises in blood pressure: toxemia of pregnancy, renal disease, pheochromocytoma, and hypertensive emergency [8,7]. However, no published reports of incidence rates among patients with these systemic diseases exist in the literature. We report the case of a patient with hypertensive emergency whose primary symptom was vision loss due to hypertensive chorioretinopathy.

## Case presentation

A 42-year-old man with no known past medical history presented with 3 weeks of blurry vision in the right eye. He had no other ocular, medical, or surgical history. He rarely sought medical care and was on no medications. Upon further history taking, he endorsed bitemporal headaches over the past 2 weeks and occasional warmth in his face over the same time period.

**Figure 1:**
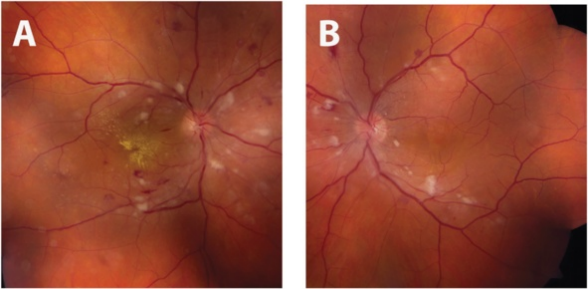
Initial fundus exam. Fundus photos of the right **(A)** and left **(B)** eye upon presentation demonstrating bilateral arteriolar narrowing, peripapillary and macular cotton-wool spots and dot-blot and flamed shaped intraretinal hemorrhages. Intraretinal exudates in the form of a macular star are also visible on the right.

**Figure 2:**
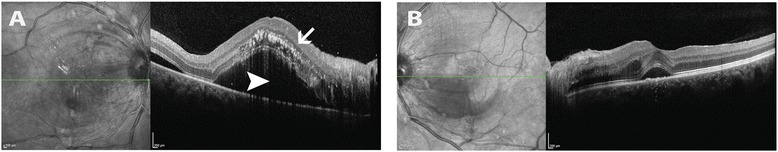
Initial retinal cross sections with optical coherence tomography (OCT). OCT images of the right **(A)** and left **(B)** eyes. The images on the left represent infrared reflectance scans of the retina, the line through this image represents the cross section of retina displayed to the right of each image. Image A demonstrates diffuse intraretinal exudates (arrows) in the right eye as well as a large serous retinal detachment at the level of the fovea and including the entire macula (arrowhead). Image B demonstrates mild subfoveal, serous retinal detachment and comparatively more normal appearing intraretinal architecture when compared to the right.

Ophthalmic examination revealed visual acuity of count fingers in the right eye and 20/20 in the left eye. His intraocular pressure was normal (19 mmHg) in both eyes. His pupils reacted normally without an afferent pupillary defect. He had normal confrontation visual fields, extraocular motility, and ocular alignment in both eyes. Slit lamp examination revealed normal anterior structures in both eyes. Retinal exam on the right revealed normal optic disc, arteriolar attenuation, diffuse macular and peripapillary cotton wool spots, intraretinal hemorrhages throughout the macula and retinal periphery, intraretinal exudation and macular star formation (Figure 1), and serous retinal detachment of the macula seen clinically and with optical coherence tomography (OCT) (Figure 2). Examination of the left eye revealed similar retinal findings (Figure 1) with lack of exudation and less significant serous retinal detachment also seen on OCT (Figure 2). Fluorescein angiography was performed and demonstrated early hyperfluorescent spots in both eyes with leakage in the late phases. The patient’s blood pressure was checked and found to be 256/160 mmHg. He was diagnosed with hypertensive emergency with hypertensive chorioretinopathy concerning for end-organ damage. He was transferred to the emergency department for urgent evaluation.

Upon arrival to the emergency department, his vital signs demonstrated normal body temperature (36.7), respiratory rate (16), and oxygen saturation (96%). In addition to his elevated blood pressure, his heart rate was also elevated to 100 beats per minute. His general physical examination was otherwise normal. Laboratory testing revealed creatinine of 2.73 with protein and casts in his urine. His electrocardiogram showed normal sinus rhythm with left ventricular hypertrophy, left atrial enlargement, and T-wave inversion in all leads except V1-V3. His troponin level was elevated (0.7). He was treated with three doses of intravenous labetolol 20 mg injection over the course of 3 h, and his blood pressure was reduced to 160/100 mmHg. He was admitted to the general medicine service with a diagnosis of hypertensive emergency with ophthalmic, cardiac, and renal end-organ damage.

His hospital course was uncomplicated and his evaluation for secondary causes of hypertension unremarkable. He was started on oral anti-hypertensive medications for essential hypertension in a step-wise fashion. He was discharged 6 days after admission on the following medications: amlodipine 10 mg daily, chlorthalidone 25 mg daily, hydralazine 25 mg daily, losartan 100 mg daily, and metoprolol 50 mg twice daily.

**Figure 3:**
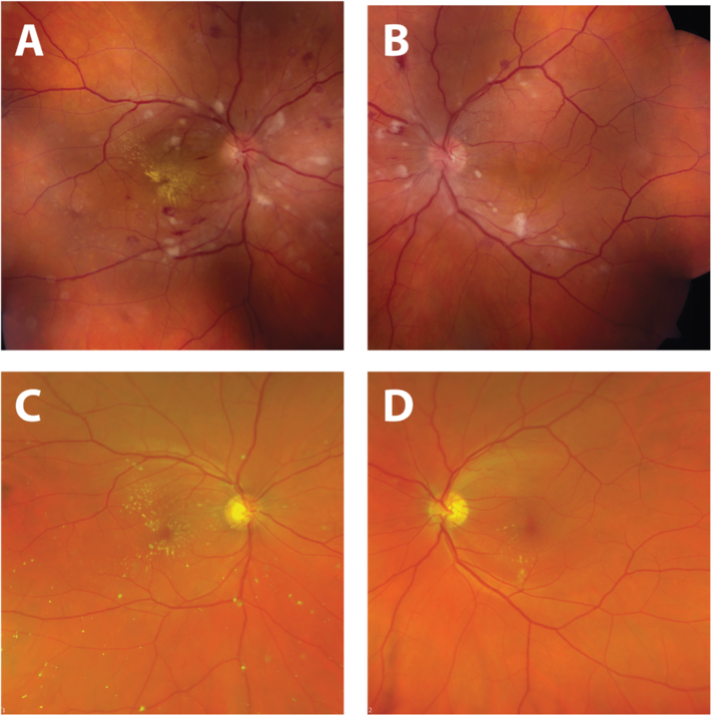
Improvement in fundus appearance after treatment. Comparison of fundus photographs taken at time of presentation **(A, B)** and 4 months after diagnosis and treatment of systemic hypertension **(C, D)**. Normalization of blood pressure resulted in resolution of retinal hemorrhages, cotton-wool spots, and serous macular detachment. There is interval improvement in hard exudates in the right eye with residual macular star.

**Figure 4:**
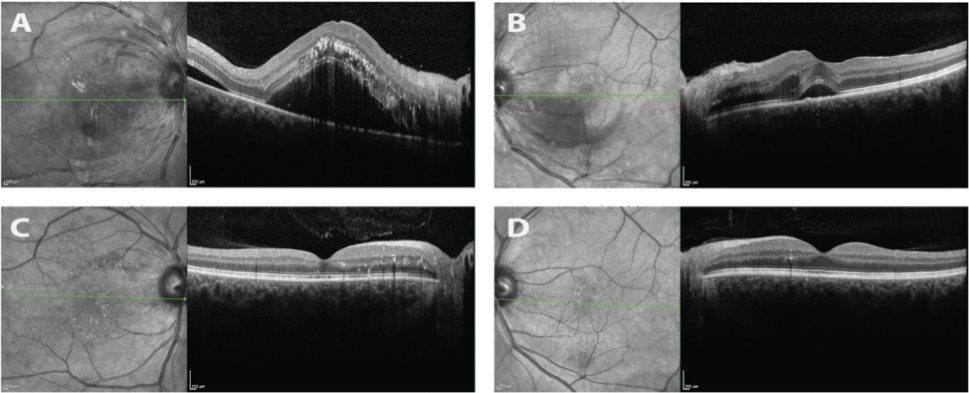
Improvement in retinal detachment after treatment. Comparison of optical coherence tomography (OCT) images of the macula at the time of presentation **(A, B)** and 4 months after treatment of systemic hypertension **(C, D)**. Normalization of blood pressure resulted in resolution of serous macular detachments in both eyes and improvement in intraretinal exudation.

Four months after discharge, the patient was compliant with all five anti-hypertensive medications and his blood pressure was controlled in the 140/90 mmHg range. His follow-up ophthalmic exam at this time revealed improved visual acuity of 20/25 in the right eye and 20/20 in the left eye. His retinal examination had improved dramatically in both eyes (Figure 3), demonstrating the resolution of cotton wool spots, retinal hemorrhages, and serous macular detachments (Figure 4).

## Conclusions

Hypertensive retinopathy is a common complication of systemic hypertension. Hypertensive choroidopathy is a less-common complication of systemic hypertension but can be the harbinger of a potentially life-threatening hypertensive emergency with end-organ damage. Choroidal involvement in the setting of hypertensive emergency is usually the sign of an acute, dramatic increase in systemic blood pressure in a young person. It can be the presenting sign of undiagnosed hypertensive emergency or even a result of potentially life-threatening, secondary causes of systemic hypertension.

This case demonstrates the important connection between system hypertension and vision complaints. An ocular fundus exam is imperative in any patient with concomitant visual symptoms and elevated blood pressure. The exam findings associated with hypertensive retinopathy can usually be seen with direct ophthalmoscopy of the undilated pupil. However, the findings associated with hypertensive choroidopathy are more subtle and a dilated exam is important in anyone with vision complaints and markedly elevated blood pressure. With the recent introduction of portable, nonmydriatic fundus cameras in the emergency departments, the incidence of detecting ocular fundus findings in hypertensive patients will increase rapidly [9]. Proper identification of hypertensive choroidopathy can add prognostic value and may aid in elucidating the time course of patients’ hypertension.

## Consent

Written informed consent was obtained from the patient for publication of this case report and any accompanying images. A copy of the written consent is available for review by the Editor-in-Chief of this journal.
